# Own Experience in Treatment of Patients with Penile Cancer Using Photodynamic Therapy

**DOI:** 10.1155/2015/245080

**Published:** 2015-03-05

**Authors:** Elena Filonenko, Andrey Kaprin, Boris Alekseev, Antonina Urlova

**Affiliations:** P.A. Herzen Moscow Cancer Research Institute, 2nd Botkinskiy Proezd 3, Moscow 125284, Russia

## Abstract

Penile cancer is a rare pathology. For penile cancer surgical treatment, radiotherapy, chemotherapy, and combined modality treatment are available. Because of great importance of this organ for mental condition of patient, the development of organ-preserving methods allowing to minimize impact on patient's quality of life without compromising of oncological results is desirable. In the Center of Laser and Photodynamic diagnosis and treatment of tumors in P.A. Herzen Moscow Cancer Research Institute the methods of photodynamic therapy in patients with penile cancer have been developed. From 2011 to 2013 the treatment was conducted in 11 patients with precancer and cancer of penile. The average age was 56.6. According to morphological diagnosis photodynamic therapy (PDT) was performed using two methods. One method included topical application of agent for PDT and the second intravenous administration of photosensitizer. For topical application alasens was used and for intravenous injection we applied radachlorine. All patients had no complications. Complete regression was achieved in 9 patients, and partial regression in 2. Thus, the results showed that photodynamic therapy for penile cancer stage Tis-1N0M0 permits performing organ-preserving treatment with satisfactory oncological results and no impairment of patient's quality of life.

## 1. Introduction

Penile cancer is a rare pathology [1]. In 2012 in Russia 493 new cases were registered [2]. The average age of patients accounts for 62.3. Increase of incidence occurs from 45 years with maximal rates in patients of 60–64 years old. For penile cancer surgical treatment, radiotherapy, chemotherapy, and combined modality treatment are available. Due to its rarity and the consequent lack of randomized trials, current therapy is based on retrospective studies and small prospective trials [3]. Surgical treatment includes resection, amputation at the level of pubic symphysis with perineal urethrostomy, emasculation, degloving, and Ducuing surgery for regional lymph node metastases. Penile amputation with following reconstruction becomes widespread. A partial and glans-sparing penectomy provides psychosocial benefits, preserves sexual function, and is generally feasible for a T1 tumor [4]. Total penectomy is preferred for ≥T2 tumors, although some T2 tumors are amenable to partial penectomy based on location. Penile-sparing surgical modalities including Mohs' micrographic surgery and laser ablation are considered for small tumors, particularly if located on the glans and margins ≥3 mm can be attained [5]. Radiotherapy is used as option of organ-preserving treatment. Chemotherapy is performed in combination with other methods. For radiotherapy there is a high risk of radiation-induced effects; surgery is also associated with risk of postoperative complications. Because of great importance of this organ for mental condition of patient, the development of organ-preserving methods allowing to minimize impact on patient's quality of life without compromising of oncological results is desirable. Penile preservation is superior in functional and cosmetic outcomes and should be offered as a primary treatment modality in men with low stage penile cancer [6].

## 2. Material and Methods

In the Center of Laser and Photodynamic diagnosis and treatment of tumors in P. A. Herzen Moscow Cancer Research Institute the methods of photodynamic therapy in patients with penile cancer have been developed. From 2011 to 2013 the treatment was conducted in 11 patients with precancer and cancer of penile. The age of patients accounted for 25 to 74 y.o. The average age was 56.6 (Table 1).

Prior to photodynamic therapy all patients had biopsy of penile lesions. According to histological type of tumor there were the following diagnoses: dysplasia grade III was in 1 patient, erythroplasia of Queyrat in 4, squamous cell carcinoma in 5 (stage T1N0M0 in 4, T2N0M0 in 1), and Paget's cancer of root of penis and scrotal skin in 1 (Table 2).

Erythroplasia of Queyrat (*n* = 2 patients) and squamous cell carcinoma of glans penis stages I-II (*n* = 2 patients) were diagnosed at first presentation in 4 patients; in the other 7 patients the interval between first presentation and accurate diagnosis accounted for up to 1 year (in 5 patients), up to 2 (in 1), and 3.5 (in 1). All 7 patients underwent nonefficient treatment with ointment by dermatologists or urologists with no morphological diagnosis during the interval. All patients had negative inguinal lymph nodes.

Primary, untreated tumor occurred in 9 patients, continued growth after chemoradiotherapy in 2, and recurrence 1 year after circumcision in 1. Eight patients had single lesion and in 3 from 2 to 3 lesions.

In 2 patients with continued growth after chemoradiotherapy, one had 1 tumor lesion 2.5 cm in size and with area of 3.75 cm^2^; the second had 3 tumor lesions with maximal size of 1.8 cm and total area of 4.5 cm^2^. One patient with recurrent tumor after circumcision had a single lesion with maximal size of 4.0 cm and area of 8 cm^2^.

For group of patients (*n* = 6) with previously untreated single tumor, in 3 patients maximal size of lesion was from 1 to 2 cm and in 3 from 3.0 to 4.5 cm. The area of tumor accounted for 1 cm^2^ to 1.5 cm^2^ in 2 patients; from 3 to 4.5 cm^2^ in 1; and from 7 to 11.3 cm^2^ in 3. For group of patients (*n* = 2) with previously untreated 2 and 3 lesions, maximal size of the largest lesion was 3.0 and 4.0 cm and total area of all lesions 10.4 cm^2^ and 7 cm^2^, respectively (Table 3).

In 9 patients penile lesion was the only cancer disease and in 2 one of primary multiple metachronous oncological processes. One of them had previous successful treatment for Kaposi's sarcoma and another one prostate cancer stage II.

Seven of 11 patients had various comorbidities: chronic obstructive lung disease, previous myocardial infarction and postinfarction cardiosclerosis, asthma, essential hypertension 1 degree, asthma combined with gastric ulcer, multiple hepatic haemangioma and bilateral renal cysts, chronic gastritis, renal cysts, and nerve deafness.

According to morphological diagnosis photodynamic therapy (PDT) was performed using two methods. One method included topical application of agent for PDT and the second intravenous administration of photosensitizer. For topical application alasens was used (agent based on 5- aminolevulinic acid) (NIOPIK, Russia) and for intravenous injection we applied radachlorine (photosensitizer based on chlorine e6) (Radapharma, Russia). After the completion of exposure time specific for each photosensitizer (for alasens-induced PPIX and for radachlorine—3 h), a session of fluorescence diagnostics with evaluation of area of tumor and planning of laser irradiation fields was performed.

**Figure 1:**
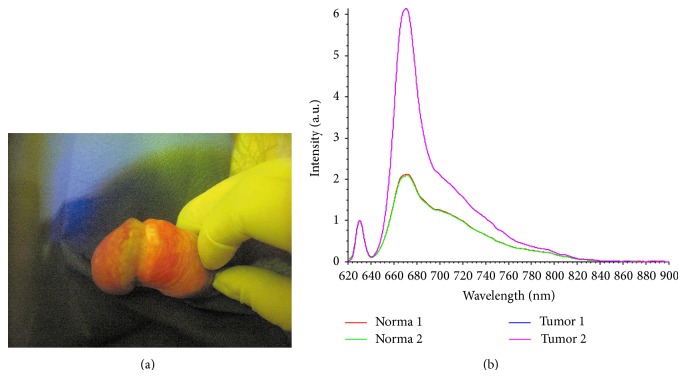
Session of fluorescence diagnosis: (a) visual assessment of fluorescence image; (b) local fluorescent spectroscopy (red curve—tumor fluorescence profile; green—normal tissue).

Fluorescence diagnosis was performed by visual assessment and by local fluorescence spectroscopy. For visual assessment of fluorescence image video-assisted fluorescence device (BioSpec, Russia) was used. After registration the fluorescence image was recorded in computer for subsequent analysis of type and boundaries of tumor lesion for planning of laser fields for PDT [7]. After visual assessment of fluorescence image local fluorescence spectroscopy was performed using laser electronic spectrum analyzer for fluorescence diagnosis LESA-01-BioSpec (BioSpec, Russia). Accumulating levels of photosensitizer in tumor and normal tissue and value of tumor/normal tissue fluorescence contrast were measured (Figure 1).

**Figure 2:**
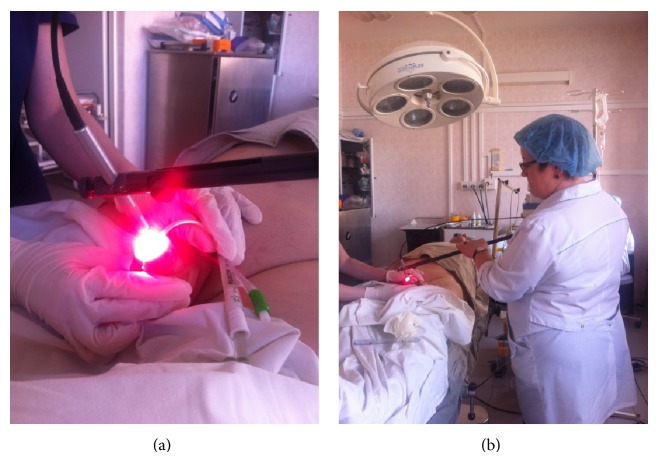
PDT session (a, b).

Then PDT session was performed. In all cases laser irradiation of tumor was performed after premedication and spinal anaesthesia. Laser irradiation was performed using macrolenses and lasers with wavelength according to peak of photosensitizer absorption: for alasens-induced PPIX—630 nm and for radachlorine—662 nm (Figure 2). The light dose accounted for 200 to 350 J/cm^2^. To prevent urinary retention because of edema in the treatment area urinary catheter was placed in all patients and was removed within 1-2 days after treatment.

## 3. Results 

All patients had no complications. There were no urinary retention after removal of catheter and no complications related to skin photosensitivity.

Complete regression was achieved in 9 patients (all 8 patients with primary tumor and 1 with recurrence) and partial regression in 2. Two patients with recurrent tumors after external radiotherapy and partial regression after PDT underwent surgical treatment 3 months after PDT.

In other 9 patients the follow-up period was from 5 to 32 months. All patients underwent physical examination, biopsy of lesion, ultrasonography of penis, and regional lymph nodes every 3 months for the first year, every 6 months for the second year, and then annually. Recurrence occurred 20 months later in PDT area in one patient who had first PDT for recurrent tumor after surgical treatment. Another patient had recurrence of disease as new lesion beyond PDT area which was diagnosed and successfully treated with PDT 9 months after first course of treatment. For 11 months the patient had no recurrence after second course of PDT.

## 4. Conclusion

Thus, photodynamic therapy for penile cancer stage Tis-1N0M0 permits to perform organ-preserving treatment with satisfactory oncological results with no impairment on patient's quality of life. We suggest that photodynamic therapy should be recommended for Tis tumors. Because of small case number our recommendations for photodynamic therapy in patients with T1 tumors require further investigations.

## Figures and Tables

**Table 1:** Patients' distribution according to age.

Number of patients	Age of patients, y.o.						Total
	20–30	31–40	41–50	51–60	61–70	71–80	
Abs.	1	0	3	2	3	2	11
%	9.0	0	27.3	18.2	27.3	18.2	100%

**Table 2:** Patients' distribution according to histological type of tumor.

Morphological diagnosis	Number of patients	
	Abs.	%
Dysplasia grade III	1	9.1
Erythroplasia of Queyrat	4	36.4
Squamous cell carcinoma T1-2N0M0	5	45.4
Paget's cancer	1	9.1
Total	**11**	**100%**

**Table 3:** Patients' distribution according to tumor characteristics and lesion area.

Tumor characteristics	Area of lesion, cm^2^			Total, abs. (%)
	≤1.5	3–4.5	7–11.3	
Untreated tumor	2	1	5	8 72.7
Recurrent/residual tumor	0	2	1	3 27.3
Total, abs. (%)	**2 (18.2%)**	**3 27.3**	**6 54.5**	**11 (100%)**
